# Benzophenone Rhamnosides and Chromones from *Hypericum seniawinii* Maxim.

**DOI:** 10.3390/molecules27207056

**Published:** 2022-10-19

**Authors:** Jing Xia, Bo Hu, Mengyu Qian, Jiayue Zhang, Lin Wu

**Affiliations:** Inflammation and Immune Mediated Diseases Laboratory of Anhui Province, School of Pharmacy, Anhui Medical University, Hefei 230032, China

**Keywords:** *Hypericum seniawinii*, Hypericaceae, benzophenone rhamnosides, anti-inflammatory activity, neuroprotective activity

## Abstract

Two new benzophenone glycosides, hypersens A and B, along with four known compounds, (*S*)-(+)-5,7-dihydroxy-2-(1-methylpropyl) chromone (**3**), 5,7-dihydroxy-2-isopropylchromone (**4**), urachromone B (**5**), and 3-8′′ bisapigenin (**6**), were isolated from *Hypericum seniawinii*. The structures of new compounds (**1** and **2**) were elucidated according to comprehensive spectroscopic data analyses. The absolute configurations of **1** and **2** were determined by electronic circular dichroism (ECD) calculations. All isolated compounds were evaluated for their neuroprotective effect using corticosterone-induced PC12 cell injury. In addition, compounds **1**–**6** were evaluated for their anti-inflammatory activity in lipopolysaccharide-induced RAW 264.7 cells. Compound **6** was a biflavonoid and significantly inhibited the production of nitric oxide with an IC_50_ value of 11.48 ± 1.23 μM.

## 1. Introduction

*Hypericum seniawinii* Maxim. (Hypericaceae) is a perennial herbaceous plant, and widely distributed in temperate regions [1]. It has been used as a folk medicine for the treatment of inflammation and infectious diseases in China [2]. Previous studies on the chemical constituent and bioactivities of this herb are minimal. However, the genus *Hypericum*, consisting of about 500 species, is an important resource of medicinal and cosmetic plants [3]. The genus *Hypericum* has been reported to contain phloroglucinol derivatives, flavonoids and xanthonoids [4,5,6,7], and other miscellaneous compounds [8] which exhibit various pharmacological activities, such as antioxidative, antitumor, antiviral, anti-inflammatory, and antifungal activities [9,10].

As a part of our investigation on bioactive compounds from natural sources [11,12,13], phytochemical investigation on this species was conducted. As a result, two new benzophenone glycosides (**1** and **2**), along with four known compounds (**3**–**6**), were isolated and characterized from *H. seniawinii*. Benzophenones glycosides isolated from the genus *Hypericum* have been reported to show various activities, such as antibacterial and anti-inflammatory activities [14]. The anti-inflammatory and neuroprotective activities of all compounds were investigated. Compounds **1**–**6** exhibited neuroprotective effects against corticosterone-induced PC12 cell injury. Moreover, all compounds reduced the lipopolysaccharide (LPS)-induced production of nitric oxide (NO) at the concentration of 10 μM. Among these, **6** showed a significant inhibitory effect with an IC_50_ value of 11.48 ± 1.23 μM.

## 2. Results and Discussion

**Figure 1:**
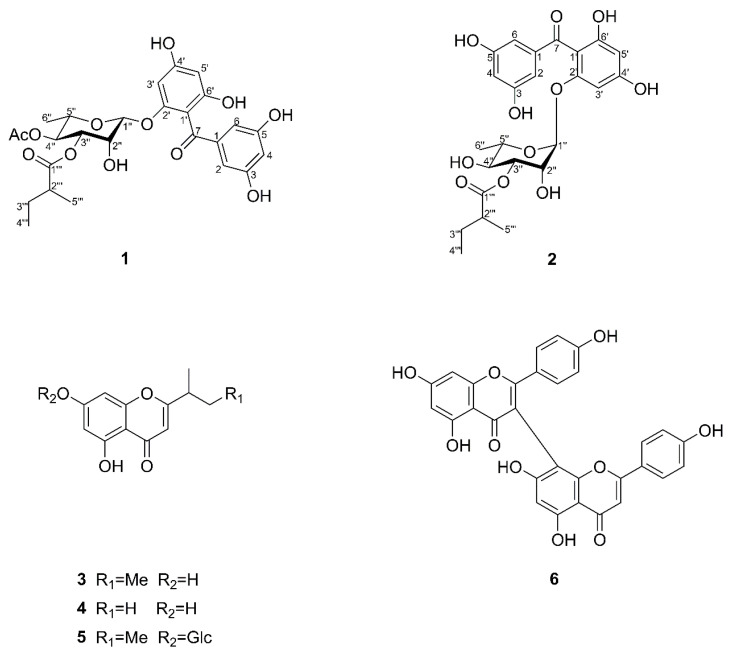
Structures of compounds **1**–**6**.

**Figure 2:**
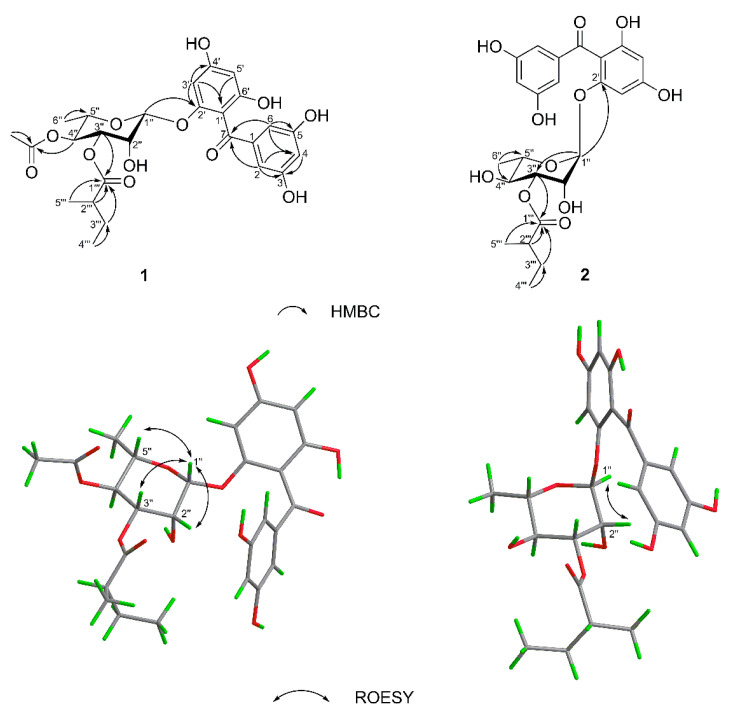
Key 2D correlations of **1** and **2**.

**Figure 3:**
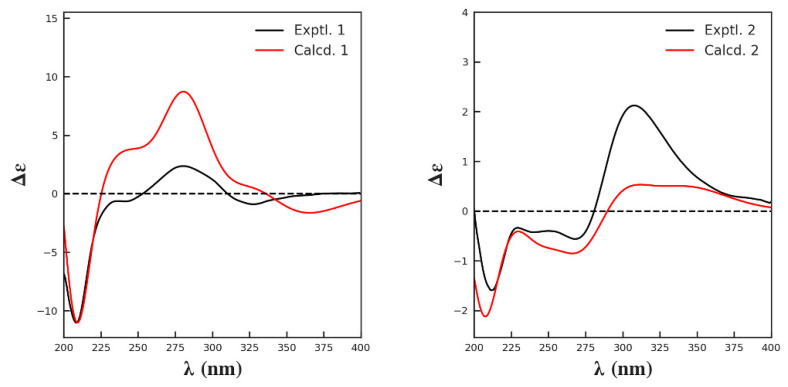
Experimental and calculated ECD spectra of **1** and **2**.

Hypersen A (**1**) was obtained as yellow gum. Its molecular formula was established as C_26_H_30_O_12_ based on its HR-ESI-MS (Figures S1–S3, Supplementary Materials) ion at 535.1776 [M + H]^+^ (calcd 535.1810), indicating 12 sites of unsaturation. The ^1^H NMR data (Table 1) showed a set of 1,3,5-trisubstituted benzene ring signals (*δ*_H_ 6.61 (2H, d, *J* = 2.3 Hz), 6.38 (1H, t, *J* = 2.3 Hz)), a 1,2,4,6-tetrasubstitutedbenzene ring (*δ*_H_ 6.23 (1H, brs) and 6.08 (1H, d, *J* = 2.1 Hz)), and characteristic resonances of a rhamnopyranosyl moiety (Figure 1) at *δ*_H_ 5.28 (1H, d, *J* = 2.0 Hz), and 1.09 (3H, d, *J* = 6.3 Hz). In addition, the ^1^H and ^13^C NMR spectra (Figures S4 and S5) revealed the presence of an acetyl group (*δ*_H_ 2.01 (3H, s), *δ*_C_ 171.9 and 20.9), and a 2-methylbutyryl group (*δ*_H_ 2.35 (1H, m), 1.46 (1H, m), 1.61 (1H, m), 0.88 (3H, t, *J* = 7.5 Hz) and 1.10 (3H, d, *J* = 6.3 Hz)). The presence of a carbonyl (*δ*_C_ 199.2) and the HMBC (heteronuclear multiple-bond correlation) correlations (Figure 2) of H-6 to C-7 (*δ*_C_ 199.2), and H-1′′ (*δ*_H_ 5.28 (1H, d, *J* = 2.0 Hz)) to C-2′ (*δ*_C_ 158.6) suggested that **1** was a benzophenone glycoside. Comparing the NMR data (Figures S6–S8) of compound **1** with those of the known analogue petiolin G [15] reveals they have similar structures, except for additional signals due to a 2-methylbutyryl group in **1**. The 2-methylbutyryl group located at C-3′′ (*δ*_C_ 72.6) in **1** is based on the HMBC correlation from H-3′′ (*δ*_H_ 4.33 (1H, dd, *J* = 9.9, 3.3 Hz)) to C-1′′′ (*δ*_C_ 177.0). The HMBC correlation (Figure 2) of H-4′′ (*δ*_H_ 5.01 (1H, t, *J* = 9.9 Hz)) to acetoxy carbonyl carbon (*δ*_C_ 171.9) indicated that the acetoxy group was attached to C-4′′. The acidic hydrolysis of compound **1** shows that l-rhamnose was the sole sugar moiety. The β-glycosidic linkage was derived from the ROESY correlations (Figure 2) from H-1′′ (*δ*_H_ 5.28 (1H, d, *J* = 2.0 Hz)) to H-2′’ (*δ*_H_ 3.71 (1H, dd, *J* = 3.3, 2.0 Hz)), H-3′′ (*δ*_H_ 4.33 (1H, dd, *J* = 9.9, 3.3 Hz)), and H-5′’ (*δ*_H_ 3.58 (1H, m)). The relative configuration of H_3_-5′′′ (*δ*_H_ 1.10 (3H, d, *J* = 6.3 Hz)) was unable to be determined in this case, due to the flexible side chain. Thus, the absolute configuration of the sugar moiety of **1** was further confirmed by comparing its calculated ECD spectrum with that of the experimental ECD spectrum (Figure 3). Finally, the structure of **1** was established as 2′,3,4′,5,6′-pentahydroxybenzophenone-2′-*O*-(4′′-acetoxy-3′′-methylbutyrate)-β-l-rhamnoside (Figure 1).

Hypersen B (**2**) was isolated as yellow gum. Its molecular formula was established as C_24_H_28_O_11_ by HR-ESI-MS (Figures S9–S11, Supplementary Materials), with a sodium adduct molecular ion peak at *m*/*z* 515.1533 [M + Na]^+^ (calcd for [M + Na]^+^ 515.1524), which required 11 sites of unsaturation. A comparison of the NMR spectroscopic data (Figures S12–S17) (Table 1) of **2** with those of **1** indicated that they are closely related in structure. The difference was the absence of an acetyl group in **2**, which was further confirmed by the molecular formula and the chemical shift of H-4′′ (*δ*_H_ 5.01 in **1**; *δ*_H_ 3.47 in **2**). The l-form of the rhamnose moiety of **2** was assigned by the same procedure as described for **1**. The α-glycosidic linkage was determined by the ROESY cross-peaks (Figure 2) between H-1′′ (*δ*_H_ 5.16 (1H, d, *J* = 2.0 Hz)) and H-2′′ (*δ*_H_ 3.36 (1H, m)). The absolute configuration of the sugar moiety of **2** was also determined by the calculation of the ECD spectrum (Figure 3). Accordingly, the structure of **2** was determined as 2′,3,4′,5,6′-pentahydroxybenzophenone-2′-*O*-(3′′-methylbutyrate)-α-l-rhamnoside.

In addition to the new compounds **1** and **2**, four known compounds were also isolated from this plant. Their structures were identified as (*S*)-(+)-5,7-dihydroxy-2-(1-methylpropyl) chromone (**3**) [16], 5,7-dihydroxy-2-isopropylchromone (**4**) [16], urachromone B (**5**) [17], and 3-8′′ bisapigenin (**6**) [18], respectively, by comparing their NMR and specific rotation data with those in the literature.

**Figure 4:**
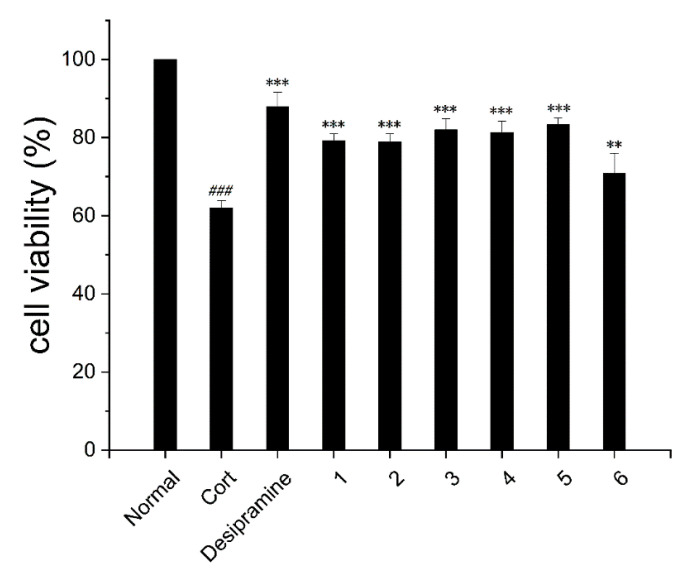
Neuroprotective effects of compounds **1**–**6** (10 μM) against CORT-induced injury in PC12 cells. Desipramine was used as the positive control (10 μM). **^###^** *p* < 0.001 vs. normal. ** *p* < 0.01, and *** *p* < 0.001 vs. desipramine-treated group.

The extracts from the genus *Hypericum* have previously been used for the treatment of depression [19]. All isolates were evaluated for their protective effects on corticosterone-induced PC12 cell injury. As shown in Figure 4, compounds **1**–**6** (10 μM) exhibited neuroprotective activity with cell viabilities of 79.27 ± 1.70%, 78.92 ± 2.09%, 82.02 ± 2.87%, 81.35 ± 2.90%, 83.35 ± 1.62%, and 70.91 ± 5.06%, respectively (62.00 ± 1.92% for the model).

**Figure 5:**
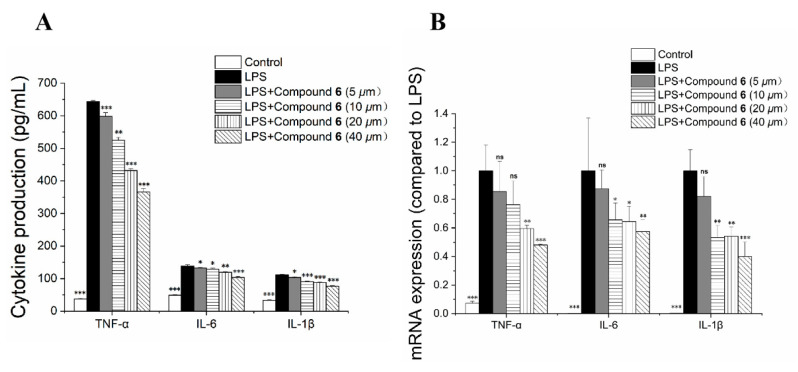
Effect of compound **6** on the production of TNF-α, IL-1β, and IL-6 (**A**), and the mRNA expression levels of TNF-α, IL-1β and IL-6 (**B**) in RAW 264.7 cells. RAW 264.7 cells were pretreated with different concentrations of compound **6** for 1 h and then stimulated with LPS (1 μg/mL) for 24 h. * *p* < 0.05, ** *p* < 0.01, and *** *p* < 0.001 vs. LPS-treated group, ns = not significant.

The anti-inflammatory activity of all isolated compounds in LPS-induced RAW 264.7 cells was investigated. The cytotoxicity of compounds **1**–**6** against RAW 264.7 cells was evaluated-using the Cell Counting Kit-8 (CCK-8) assay [20]. None of the compounds were cytotoxic to RAW 264.7 cells at a concentration of 50 μM. As depicted in Table 2, all compounds (10 μM) exhibited inhibitory effects on NO production in LPS-induced RAW 264.7 cells. Among these, compound **6** exhibited the strongest inhibitory effect with an IC_50_ value of 11.48 ± 1.23 μM. Therefore, the effect of compound **6** on tumor necrosis factor-α (TNF-α), interleukin-1β (IL-1β), and interleukin-6 (IL-6) production in LPS-induced RAW 264.7 cells was detected via enzyme-linked immunosorbent assay (ELISA). Moreover, the effect of compound **6** on mRNA expression levels of TNF-α, IL-1β, and IL-6 in LPS-induced RAW 264.7 cells was also measured. As shown in Figure 5, compound **6** inhibited the production of TNF-α, IL-1β and IL-6 by down-regulating the mRNA expressions of TNF-α, IL-1β, and IL-6.

## 3. Experimental

### 3.1. General Experimental Procedures

UV data were collected using a Shimadzu UV-1600PC spectrophotometer (Shimadzu Co., Kyoto, Japan). ECD spectra were measured on a Chirascan circular-dichroism spectrometer (Applied Photophysics Ltd., Surrey, UK). NMR spectra were recorded on a Bruker 500 spectrometer (Bruker Co., Billerica, MA, USA) with TMS as the internal standard. HRESIMS spectra were recorded on an Agilent Technologies 6224 TOF liquid chromatograph mass spectrometer instrument (Agilent Technologies, Santa Clara, CA, USA). Column chromatography (CC) was performed on silica gel (Qingdao marine Chemical Co., Ltd., Qingdao, China), ODS (40–63 μm, FuJi, Chiryu, Japan), Amberlite XAD7HP (0.56–0.71 mm, Rohm and Hass Co., Philadelphia, PA, USA), CHEETAH Flashchromatography (Agela Technologies Co., Ltd., Tianjin, China), and Sephadex LH-20 (Pharmacia, Sweden). Preparative HPLC separations were performed on a Shimadzu LC-20AR instrument (Shimadzu Co., Kyoto, Japan) using a shim-pack RP-C18 column (20 × 150 mm). Analytical and semipreparative HPLC were performed utilizing a Thermo Scientific Ultimate 3000 instrument (Thermo Fisher Scientific Inc., Waltham, MA, USA) equipped with a DAD detector, using a shim-pack VP–ODS column (4.6 × 250 mm) and a SP ODS-A column (10 × 250 mm), respectively.

### 3.2. Plant Material

The air-dried aerial parts of *Hypericum seniawinii* Maxim. (Hypericaceae) were collected from Yuexi county, Anqing city, Anhui Province, China, in June 2018. A voucher specimen (No. XJ201806) has been deposited in the School of Pharmacy, Anhui Medical University.

### 3.3. Extraction and Isolation

The air-dried aerial parts of *H. seniawinii* (500 g) were powdered and extracted with CH_2_Cl_2_. After removing the solvent, the CH_2_Cl_2_ extract (20 g) was eluted by a gradient of Petroleum ether/EtOAc (12:1 to 1:4) on a silica gel column for a total of six fractions (Fr. A–F). Fr. D (286 mg) was subjected to a silica gel column and eluted with Petroleum ether/EtOAc (10:1 to 1:2) to obtain two subfractions (Fr. D.1–2). Fr. D.1 (75.8 mg) was chromatographed over a Sephadex LH-20 column (i.d. 200 × 1.5 cm), eluting with MeOH to give four fractions (Fr. D.1.1–4). Fr. D.1.3 (5.2 mg) was further separately subjected to preparative HPLC using MeOH/H_2_O (70:30, 10 mL/min) to give compound **3** (5 mg t_R_ = 10.2 min) and **4** (1.3 mg t_R_ = 7.7 min). Fr. F (9 g) was absorbed on XAD7HP macroporon resin eluted with MeOH-H_2_O (10:90 to 90:10) to generate fractions (Fr. F.1–8). Fr. F.5 (735 mg) was then applied onto an ODS column (i.d. 30 × 3 cm) using a step gradient of MeOH/H_2_O (30:70 to 100:0) to give ten fractions (Fr. F.5.1–10). Fr. F.5.2 (55 mg) was submitted to Sephadex LH-20 column (i.d. 100 × 1.5 cm), eluting with CH_2_Cl_2_/MeOH (1:1) to obtain three subfractions (Fr. F.5.2.1–3). Fr. F.5.2.2 (15.6 mg) was purified by preparative HPLC with MeOH/H_2_O (40:60, 10 mL/min) to afford compound **2** (1.8 mg t_R_ = 15.3 min). Fr. F.5.4 (78.7 mg) was subjected to a silica gel column and eluted with CH_2_Cl_2_/MeOH (30:1 to 1:1) to give five subfractions (Fr. F.5.4.1–5). The purification of Fr. F.5.4.3 (25.8 mg) and Fr. F.5.4.5 (10.7 mg) was then subjected to preparative HPLC with MeOH/H_2_O (45:55, 10 mL/min) to obtain compounds **1** (9.9 mg t_R_ = 28.5min) and **5** (3.3 mg t_R_ = 21.6 min). Fr. F.8 (1.0265 g) was fractioned over flash chromatography eluting with MeOH/H_2_O (55:45, 10 mL/min), Fr. F.8.8 (52.4 mg) was subjected to a Sephadex LH-20 column (i.d. 100 × 1.5 cm) to yield compound **6** (12.7 mg, t_R_ = 7.6 min).

#### 3.3.1. Hypersen A (1)

Yellow gum; [α]^20^_D_ −32 (*c* 0.15, MeOH); UV (MeOH) λ_max_ (log ε) 218 (3.90), 282 (4.14), 306 (4.19) nm; ECD (MeOH) λ_max_ (mdeg) 209 (−12.54), 279 (+2.43), 329 (−0.97) nm; ^1^H NMR and ^13^C NMR data, Table 1; HR-ESI-MS *m/z* 535.1776 [M + H]^+^ (calcd for C_26_H_31_O_12_, 535.1810).

#### 3.3.2. Hypersen B (2)

Yellow gum; [α]^20^_D_ −2 (*c* 0.10, MeOH); UV (MeOH) λ_max_ (log ε) 206 (4.16), 281 (3.42), 306 (3.46) nm; ECD (MeOH) λ_max_ (mdeg) 210 (−1.71), 273 (−0.59), 308 (+2.158) nm; ^1^H NMR and ^13^C NMR data, Table 1; HR-ESI-MS *m*/*z* 515.1533 [M + Na]^+^ (calcd for C_24_H_28_O_11_Na, 515.1524).

### 3.4. ECD Calculations

According to the relative configuration of each compound deduced from the coupling constant and ROESY spectrum, systematic conformational searches were performed with Confab [21]. The initial conformations were optimized and re-optimized with the Molclus program (version 1.9.9.5) [22] by invoking the xtb program (version 6.4) [23,24] and ORCA-5.0 [25,26] at the B97-3c level. The programORCA-5.0 was used to calculate the ECD spectra at the PBE0/def2-SV(P) level with a CPCM solvent model (methanol).

### 3.5. Hydrolysis of Compounds **1** and **2**

The compound (0.5 mg) was treated with 3 M hydrochloric acid (0.5 mL) at 90 °C for 2 h. After neutralization with 3 M ammonium hydroxide, the reactants were dried by evaporation of the solvent. l-cysteine methyl ester (0.5 mg) and pyridine (0.2 mL) were added, and then stirred at 60 °C for 1 h. Finally, phenyl isothiocyanate solution (0.5 mL) was added and stirred at 60 °C for 1 h. [27]. The residue of each sample was subjected to analytical HPLC (with MeCN/H_2_O (25:75,1.0 mL/min) using a C18 RP column (4.6 mm × 250 mm, 5 μm, Thermo Fisher Scientific Inc., Waltham, MA, USA). The configuration of the sugars was determined by comparing their retention times with that of derived l-rhamnose (*t*_R_ = 20.2 min).

### 3.6. Biological Assay

#### 3.6.1. Cytotoxicity Assay

The cytotoxicity was measured by the Cell Counting Kit-8 assay (CCK-8, Beyotime, Shanghai, China). RAW 264.7 mouse macrophages were purchased from Procell Life Science & Technology Co. Ltd. (Wuhan, China, Product No. CL-0190) and PC12 (rat adrenal pheochromocytoma cell line) from the Beijing Stem Cell Bank (BSCS) (Beijing, China, Accession No. TCR 9). Both types of cells were cultured in DMEM supplemented with 10% fetal bovine serum and inoculated in 96-well plates at a density of 5 × 10^3^ cells/well. Then, the cells were treated with compounds (3.125, 6.25, 12.5, 25, 50 μM) for 24 h. CCK-8 solution (100 μL) was added to each well, and the cells were further incubated for 1.5 h. The absorbance (OD values) was measured on a microplate spectrophotometer (Synergy HTX, Biotek, Shoreline, WA, USA) at 450 nm (wavelength).

#### 3.6.2. Neuroprotective Assay

The neuroprotection was analyzed using the CCK-8 colorimetric assay. Compounds were dissolved in dimethyl sulfoxide (DMSO) (50 mM) solution. CORT (400 μM) served as an injury model group. PC12 cells were divided into no treatment (normal group), CORT (400 μM) (negative control group), CORT (400 μM) plus desipramine (10 μM) (positive control group), and CORT (400 μM) plus compounds (10 μM). Cell suspension (100 μL) was seeded in 96-well plates (5 × 10^3^ cells/well). The cells were then incubated for 24 h, and 10% CCK-8 (100 μL) medium was added to each well and incubated further for 2 h. The absorbance was measured at 450 nm using a BioTek Synergy HTX multimode reader.

#### 3.6.3. NO Production

RAW 264.7 macrophages were seeded in 48-well plates, pretreated with compounds (10 μM) for 1 h, and subsequently incubated with LPS (1.0 μg/mL) (*Escherichia coli*, Sigma-Aldrich, St. Louis, MO, USA) for 24 h. Griess reagent (Beyotime, Shanghai, China) was used to measure NO production. The absorbance was determined at 540 nm using a BioTek Synergy HTX multimode reader.

#### 3.6.4. Determination of IL-6, TNF-α, and IL-1β

The concentrations of IL-1β, IL-6, and TNF-α in the supernatants of RAW 264.7 cells were investigated by ELISA (ELISA LAB, Wuhan, China) according to the manufacturer’s protocols.

#### 3.6.5. mRNA Expressions of IL-6, IL-1β, and TNF-α

Sample RNA was extracted utilizing the triol method (Accurate Biology, Changsha, China). The RNA concentration of each sample was determined on the DS-11 Spectrophotometer (Denovix Inc., Wilmington, DE, USA) and verified for purity. cDNA synthesis was performed using a 5 × Evo M-MLV RT Master Mix kit (Accurate Biology, Changsha, China). The relative levels of selected mRNAs were measured using a SYBR Green qPCR Kit (Accurate Biology, Changsha, China) and a CFX96 Real-time RT-PCR detection system (Bio-Rad Laboratories, Inc., Berkeley, CA, USA). The mRNA expression values were normalized by internal control β-actin. Primer sequences: β-actin (forward, 5′-AGTGTGACGTTGACATCCGT-3′; reverse, 5′-TGCTAGGAGCCAGAGCAGTA-3′); TNF-α (forward, 5′-CACCACCATCAAGGACTCAA-3′; reverse, 5′-AGGCAACCTGACCACTCTCC-3′); IL-6 (forward, 5′-CTTTGAAGTTGACGGACCC-3′; reverse, 5′-TGAGTGATACTGCCTGCCTG-3′); IL-1β (forward, 5′-GAGGATACCACTCCCAACAGACC-3′; reverse, 5′-AAGTGCATCATCGTTGTTCATACA-3′).

#### 3.6.6. Statistical Analysis

IBM SPSS 25.0 software (Armonk, NY, USA) was used for the statistical analysis. Data are presented as mean ± SD (*n* = 3).

## 4. Conclusions

In summary, two new benzophenone glycosides (**1** and **2**), along with four known compounds (**3**–**6**) were isolated from the aerial parts of *H. seniawinii.* The absolute configurations of the sugar moiety of benzophenone glycosides were determined by hydrolysis and the calculation of the ECD spectrum. The neuroprotective and anti-inflammatory activities of these compounds were evaluated. Based on the results, compound **6** possessed a significant inhibitory effect on the production of NO, TNF-α, IL-1β, and IL-6 in LPS-induced RAW 264.7 cells. This study will enrich the chemical diversity of *H. seniawinii* and facilitate the development of inflammatory inhibitors and neuroprotective agents.

## Figures and Tables

**Table 1:** ^1^H (500 MHz) and ^13^C (125 MHz) NMR data for **1** and **2** in methanol-*d*_4_.

	1		2	
No.	*δ*_H_ (*J* in Hz)	*δ*_C_	*δ*_H_ (*J* in Hz)	*δ*_C_
1		143.9		144.7
2,6	6.61 (2H, d, *J* = 2.3 Hz)	107.9	6.59 (2H, d, *J* = 2.3 Hz)	108.1
3,5		159.8		159.6
4	6.38 (1H, t, *J* = 2.3 Hz)	107.7	6.37 (1H, t, *J* = 2.3 Hz)	107.6
7		199.2		199.5
1′		109.1		108.0
2′		158.6		159.7
3′	6.23 (1H, brs)	94.9	6.32 (1H, brs)	96.1
4′		164.1		165.3
5′	6.08 (1H, d, *J* = 2.1 Hz)	98.0	6.07 (1H, d, *J* = 2.1 Hz)	98.2
6′		164.0		164.4
1′′	5.28 (1H, d, *J* = 2.0 Hz)	99.5	5.16 (1H, d, *J* = 2.0 Hz)	100.8
2′′	3.71 (1H, dd, *J* = 3.3, 2.0 Hz)	69.0	3.36 (1H, m)	69.6
3′′	4.33 (1H, dd, *J* = 9.9, 3.3 Hz)	72.6	4.58 (1H, dd, *J* = 9.8, 3.3 Hz)	74.2
4′′	5.01 (1H, t, *J* = 9.9 Hz)	71.8	3.47 (1H, t, *J* = 9.8 Hz)	71.3
5′′	3.58 (1H, m)	68.8	3.55 (1H, m)	71.2
6′′	1.09 (3H, d, *J* = 6.3 Hz)	17.8	1.23 (3H, d, *J* = 6.1 Hz)	18.0
1′′′		177.0		177.7
2′′′	2.35 (1H, m)	42.1	2.42 (1H, m)	42.3
3′′′	1.46 (1H, m)	27.9	1.69 (1H, m)	27.8
	1.61 (1H, m)		1.48 (1H, m)	
4′′′	0.88 (3H, t, *J* = 7.5 Hz)	11.9	0.93 (3H, t, *J* = 7.5 Hz)	11.9
5′′′	1.10 (3H, d, *J* = 6.3 Hz)	16.6	1.16 (3H, d, *J* = 7.0 Hz)	16.9
-Ac		171.9		
	2.01 (3H, s)	20.9		

**Table 2:** Anti-inflammatory activity of compounds **1**–**6** in LPS-induced RAW 264.7 cells.

Compound	Inhibition of NO Production (%) (10 μM)	IC_50_ (μM)
1	19.21 ± 3.15	>20
2	14.55 ± 3.14	>20
3	14.26 ± 1.33	>20
4	7.57 ± 4.03	>20
5	18.92 ± 1.01	>20
6	35.51 ± 6.13	11.48 ± 1.23
Indomethacin	36.68 ± 1.33	15.04 ± 3.31

## Data Availability

The data presented in this study are available in supplementary material.

## Supplementary Materials

The following supporting information can be downloaded at: https://www.mdpi.com/article/10.3390/molecules27207056/s1. Figure S1: HRESIMS data of compound **1**; Figure S2: UV spectrum of compound **1** in CH_3_OH; Figure S3: CD spectrum of compound **1** in CH_3_OH; Figure S4: ^1^H NMR spectrum of compound **1** (500 MHz, Methanol-*d*_4_); Figure S5: ^13^C NMR spectrum of compound **1** (125 MHz, Methanol-*d*_4_); Figure S6: HSQC spectrum of compound **1** (500 MHz, Methanol-*d*_4_); Figure S7: HMBC spectrum of compound **1** (500 MHz, Methanol-*d*_4_); Figure S8: ROESY spectrum of compound **1** (500 MHz, Methanol-*d*_4_); Figure S9: HRESIMS data of compound **2**; Figure S10: UV spectrum of compound **2** in CH_3_OH; Figure S11: CD spectrum of compound **2** in CH_3_OH; Figure S12: ^1^H NMR spectrum of compound **2** (500 MHz, Methanol-*d*_4_); Figure S13: ^13^C NMR spectrum of compound **2** (125 MHz, Methanol-*d*_4_); Figure S14: HSQC spectrum of compound **2** (500 MHz, Methanol-*d*_4_); Figure S15: HMBC spectrum of compound **2** (500 MHz, Methanol-*d*_4_); Figure S16: ROESY spectrum of compound **2** (500 MHz, Methanol-*d*_4_); Figure S17: ^1^H-^1^H COSY spectrum of compound **2** (500 MHz, Methanol-*d*_4_).

## Sample Availability

Compounds were deposited in the School of Pharmacy, Anhui Medical University.
