# Supplementary material for: Benzophenone Rhamnosides and Chromones from Hypericum seniawinii Maxim
Source: Molecules. 2022 Oct 19;27(20):7056. doi: 10.3390/molecules27207056 (PMC9609419; doi:10.3390/molecules27207056)
Supplement: Supplementary file 1 [file molecules-27-07056-s001.zip › molecules-1973139-supplementary.pdf]

Supplementary Material

Benzophenone Rhamnosides and Chromones from *Hypericum seniawinii* Maxim.

Jing Xia, Bo Hu, Mengyu Qian, Jiayue Zhang, Lin Wu[*]

School of Pharmacy, Anhui Medical University; Inflammation and Immune Mediated Diseases Laboratory of Anhui Province, Anhui Medical University, Hefei 230032, China

Tel/Fax: +86-0551-65169750, E-mail: wulin@ahmu.edu.cn

The list of supplementary content

Fig. S1. HRESIMS date of compound **1**. 3

Fig. S2. UV spectrum of compound **1** in CH_3OH. 3

Fig. S3. CD spectrum of compound **1** in CH_3OH. 4

Fig. S4. ^{1}H NMR spectrum of compound **1** (500 MHz, Methanol-d_4). 4

Fig. S5. ^{13}C NMR spectrum of compound **1** (125 MHz, Methanol-d_4). 5

Fig. S6. HSQC spectrum of compound **1** (500 MHz, Methanol-d_4) 5

Fig. S7. HMBC spectrum of compound **1** (500 MHz, Methanol-d_4). 6

Fig. S8. ROESY spectrum of compound **1** (500 MHz, Methanol-d_4). 6

Fig. S9. HRESIMS data of compound **2**. 7

Fig. S10. UV spectrum of compound **2** in CH_3OH. 7

Fig. S11. CD spectrum of compound **2** in CH_3OH. 8

Fig. S12. ^{1}H NMR spectrum of compound **2** (500 MHz, Methanol-d_4). 8

Fig. S13. ^{13}C NMR spectrum of compound **2** (125 MHz, Methanol-d_4). 9

Fig. S14. HSQC spectrum of compound **2** (500 MHz, Methanol-d_4) 9

Fig. S15. HMBC spectrum of compound **2** (500 MHz, Methanol-d_4). 10

Fig. S16. ROESY spectrum of compound **2** (500 MHz, Methanol-d_4). 10

Fig. S17. ^{1}H-^{1}H COSY spectrum of compound **2** (500 MHz, Methanol-d_4). 11

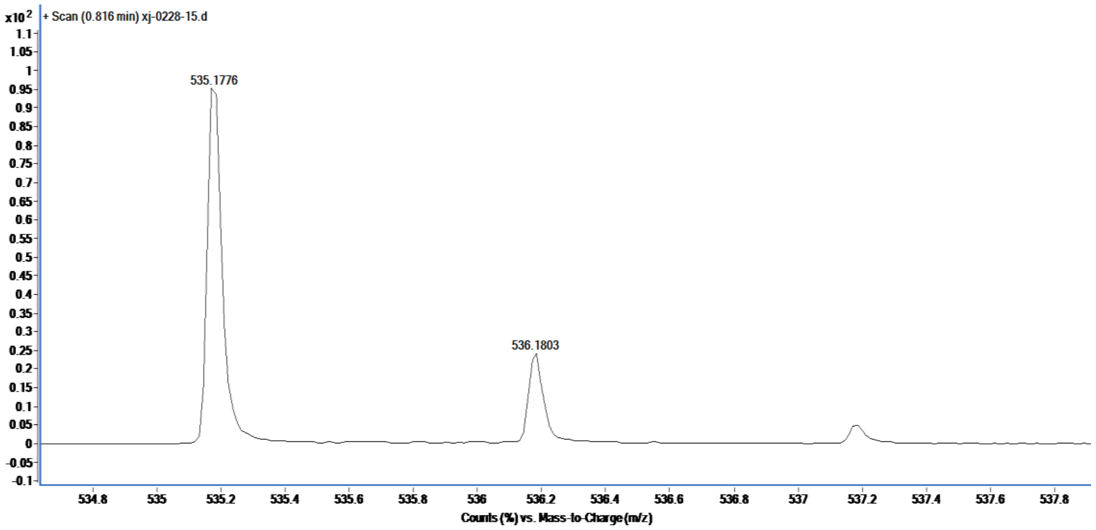

Fig. S1. HRESIMS date of compound **1**.

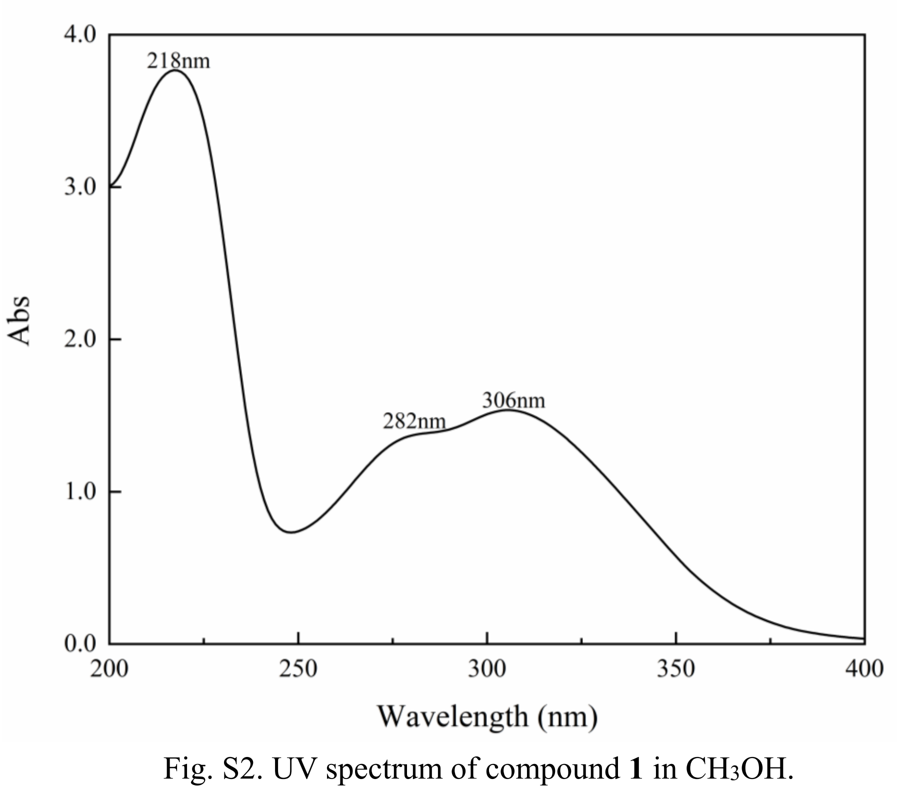

Fig. S2. UV spectrum of compound **1** in CH_3OH.

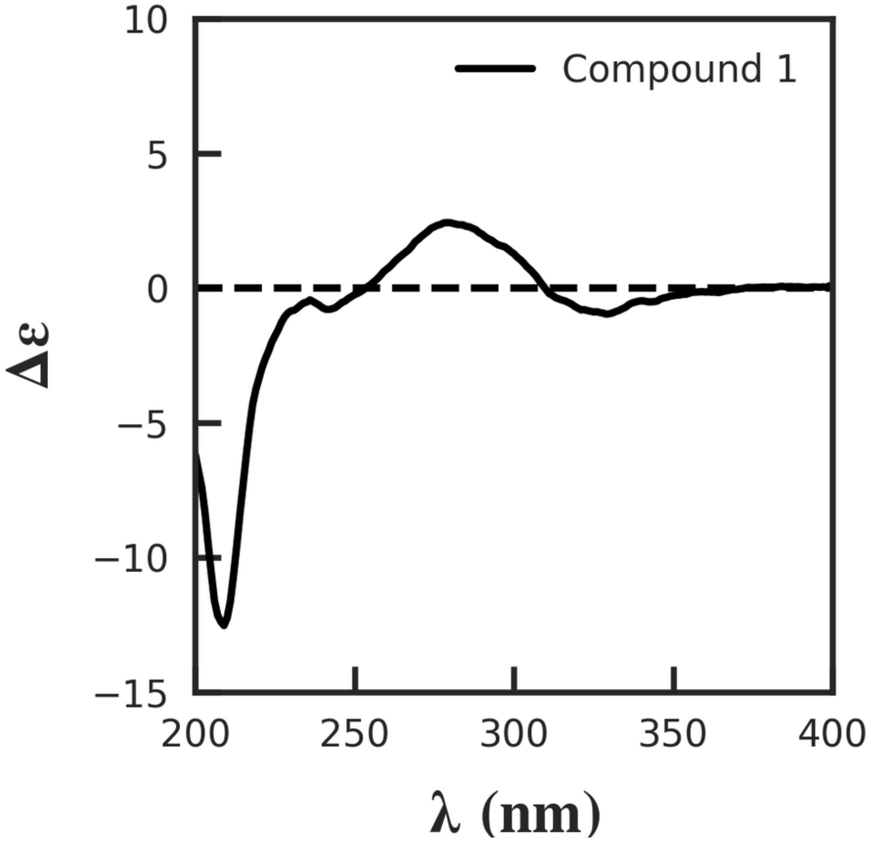

Fig. S3. CD spectrum of compound **1** in CH_3OH.

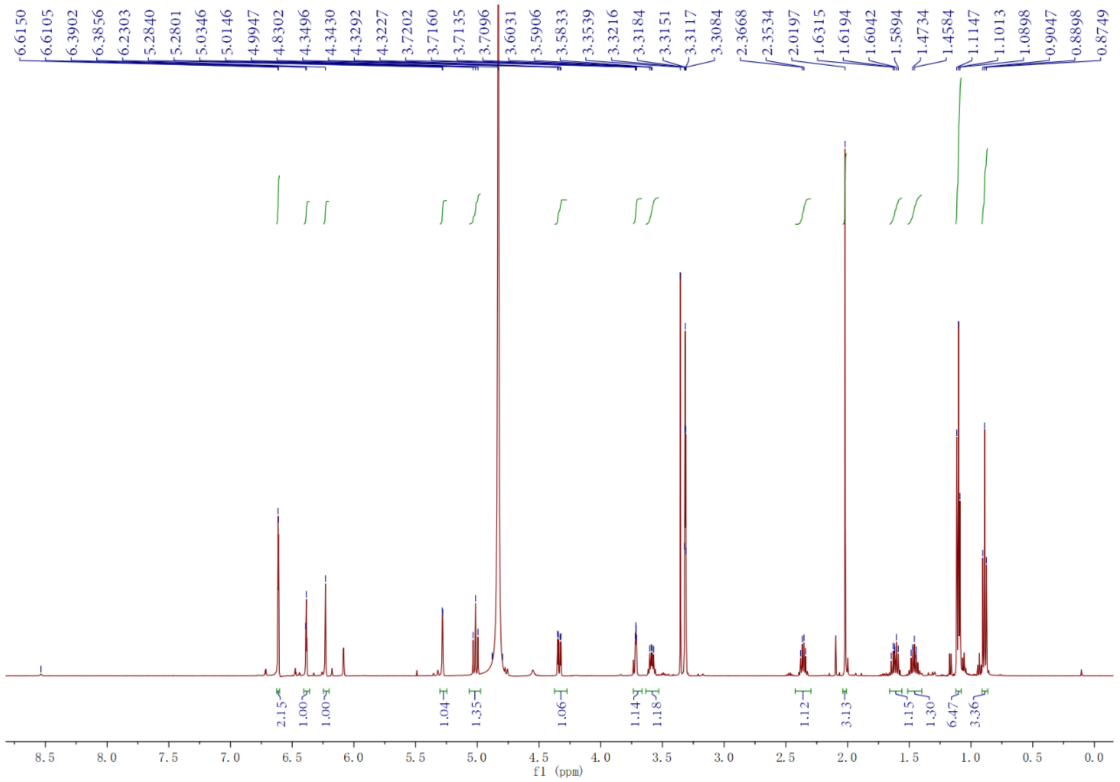

Fig. S4. ^{1}H NMR spectrum of compound **1** (500 MHz, Methanol-d_4**)**.

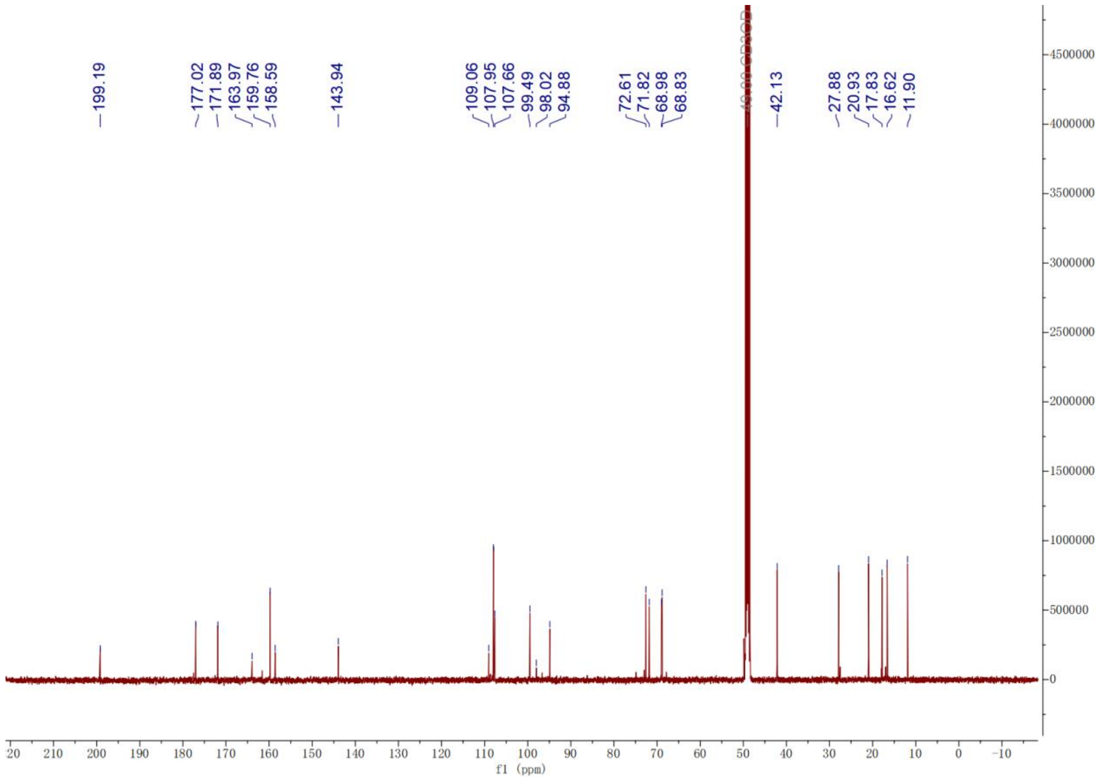

Fig. S5. ^{13}C NMR spectrum of compound **1** (125 MHz, Methanol-*d*4**).**

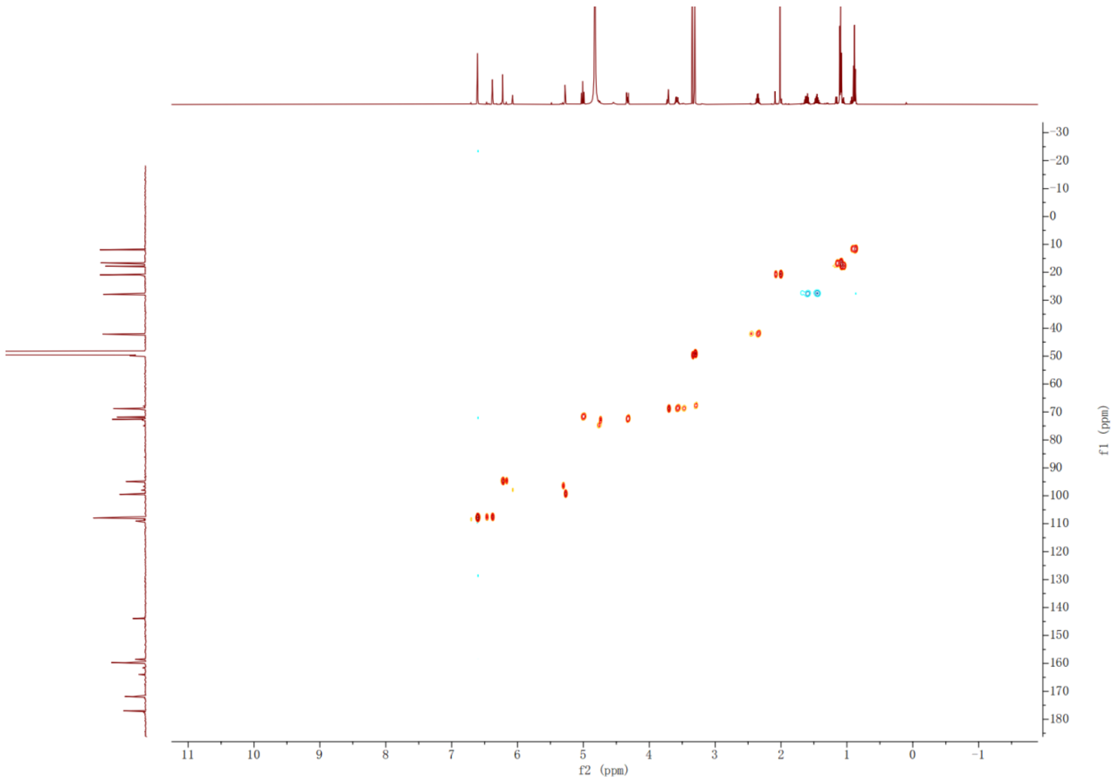

Fig. S6. HSQC spectrum of compound **1** (500 MHz, Methanol-*d*4**).**

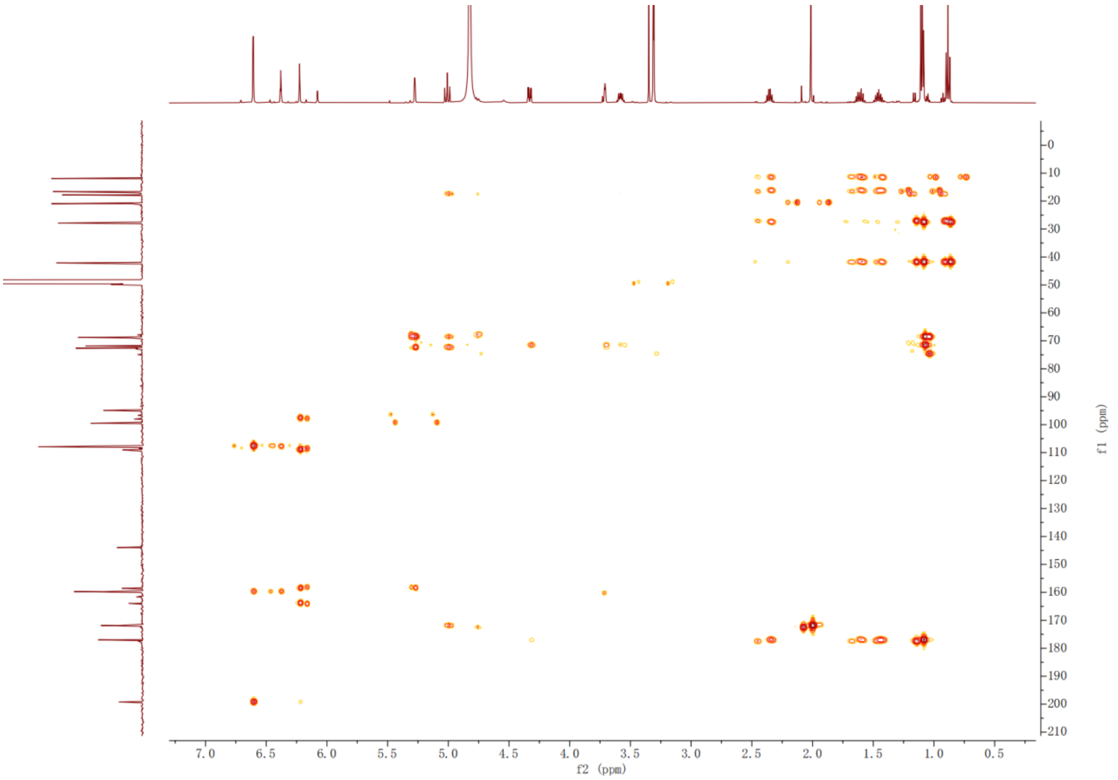

Fig. S7. HMBC spectrum of compound **1** (500 MHz, Methanol-d_4).

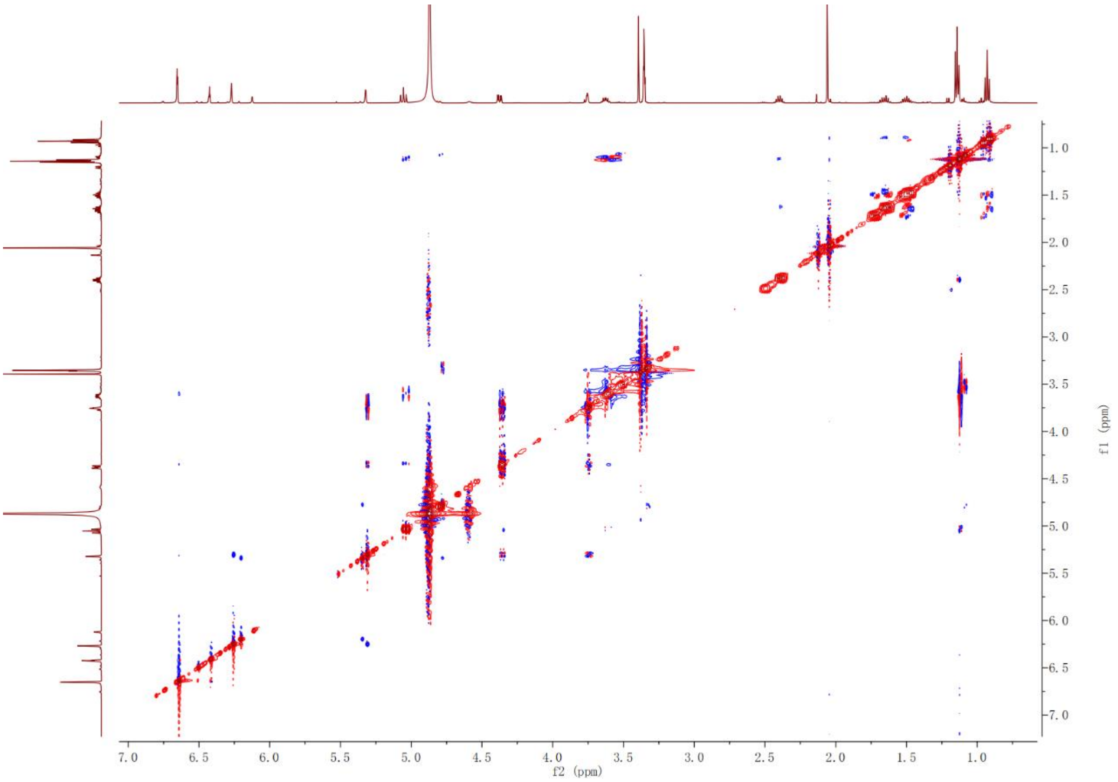

Fig. S8. ROESY spectrum of compound **1** (500 MHz, Methanol-d_4).

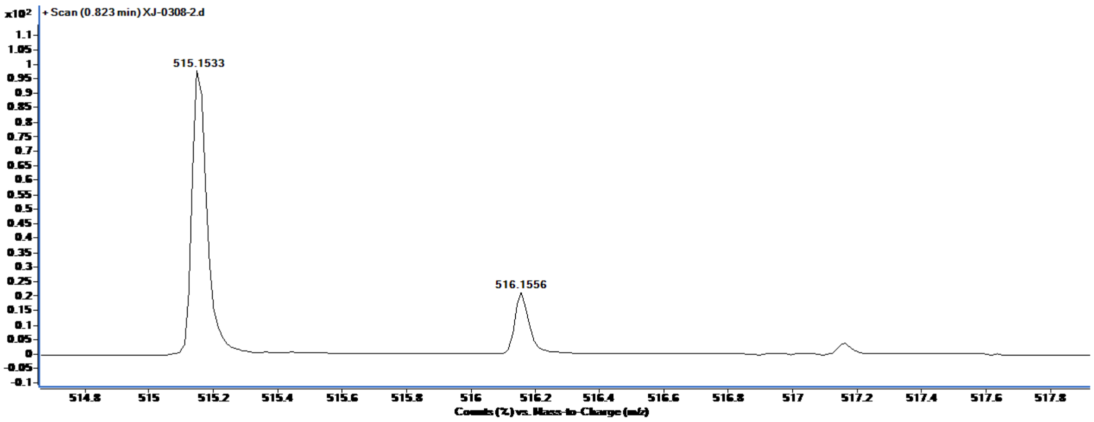

Fig. S9. HRESIMS data of compound **2**.

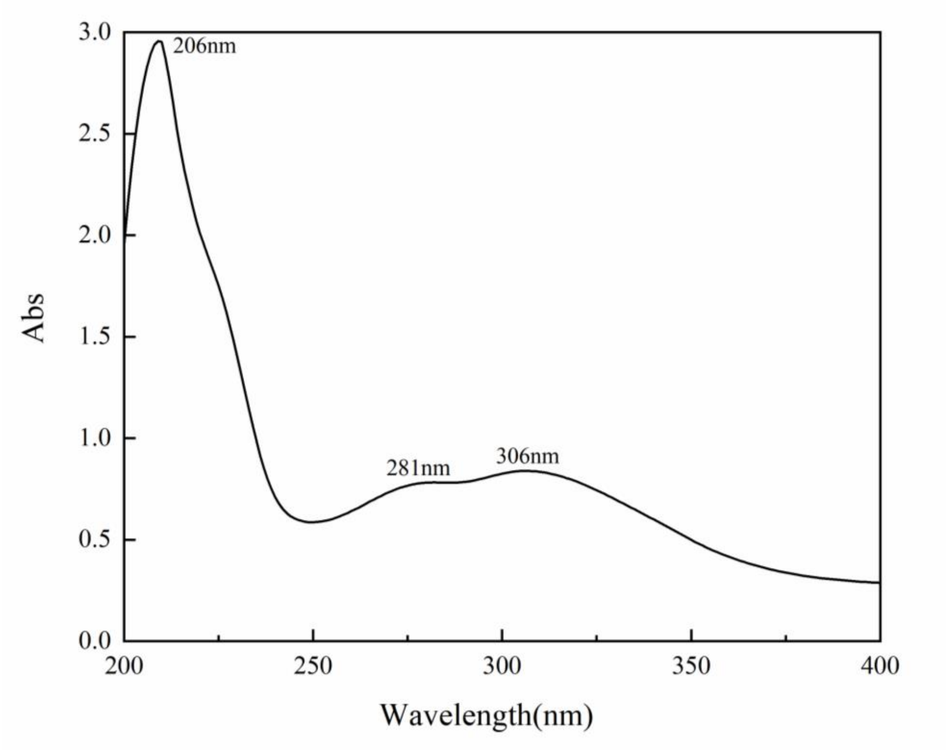

Fig. S10. UV spectrum of compound **2** in CH_3OH.

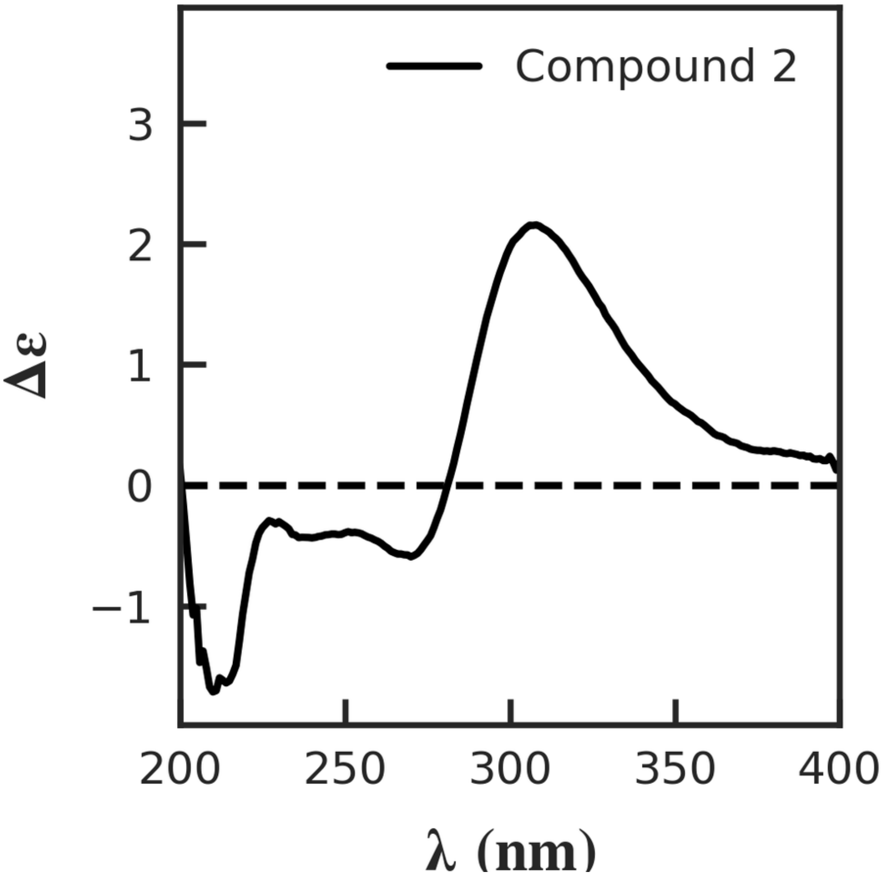

Fig. S11. CD spectrum of compound **2** in CH_3OH.

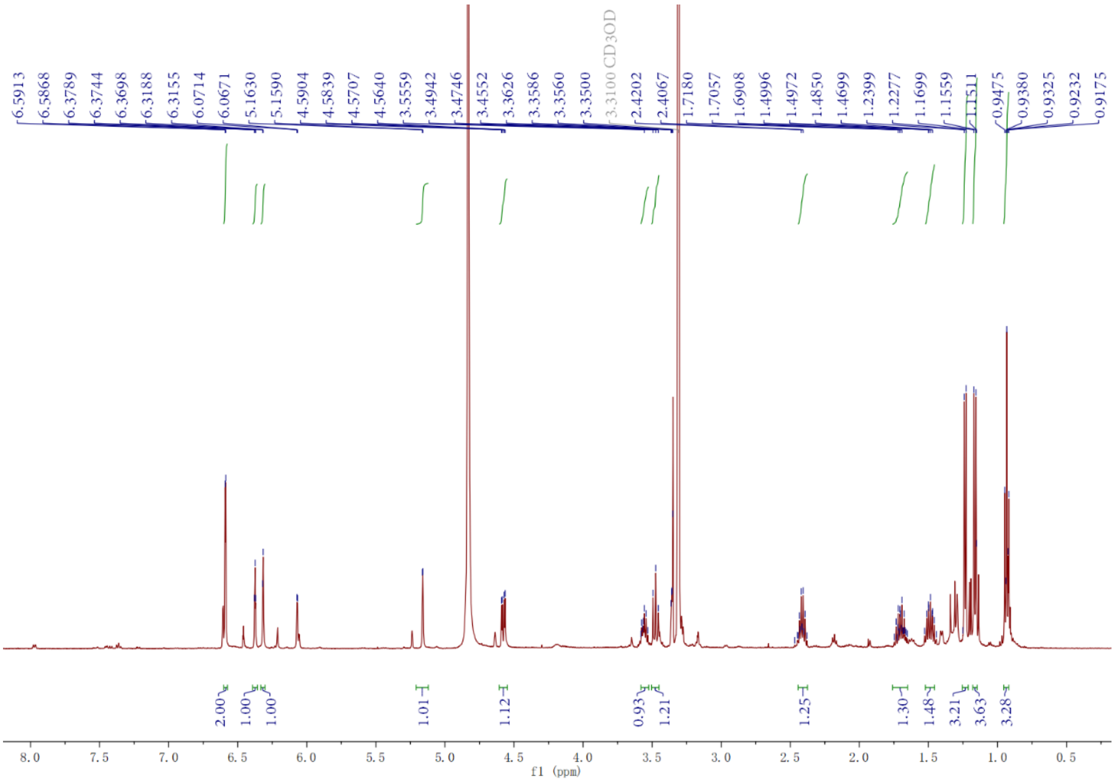

Fig. S12. ^1H NMR spectrum of compound **2** (500 MHz, Methanol-d_4).

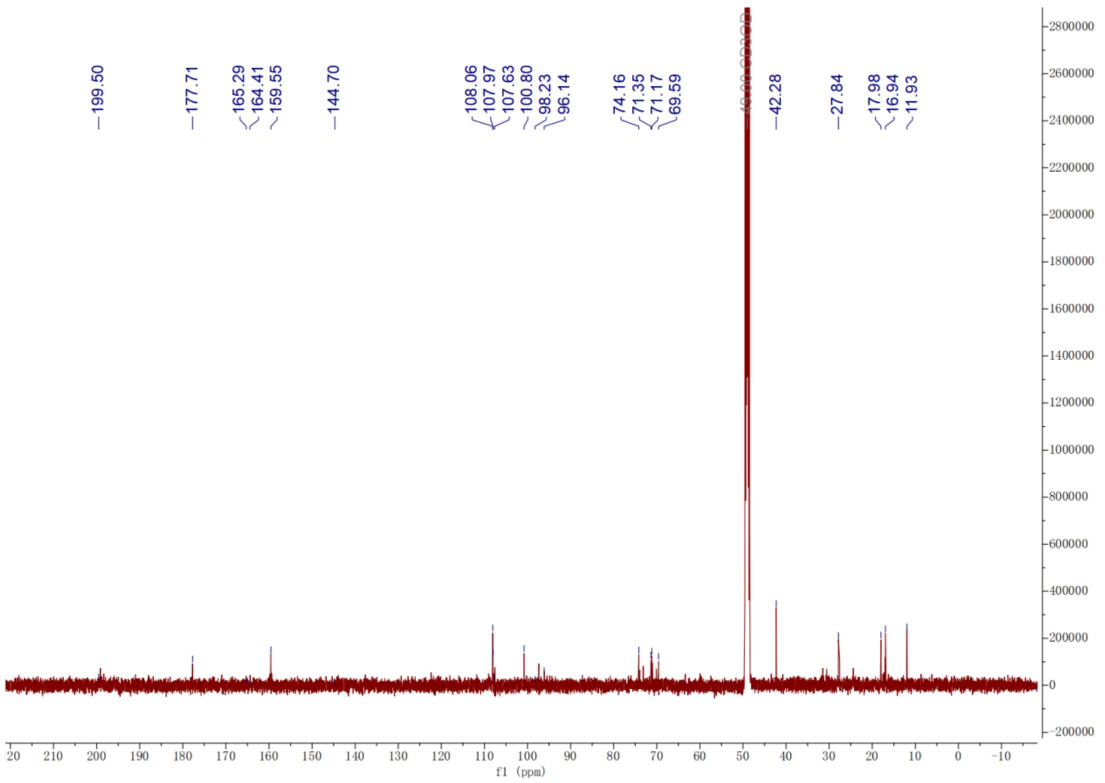

Fig. S13. ^{13}C NMR spectrum of compound **2** (125 MHz, Methanol-*d*4).

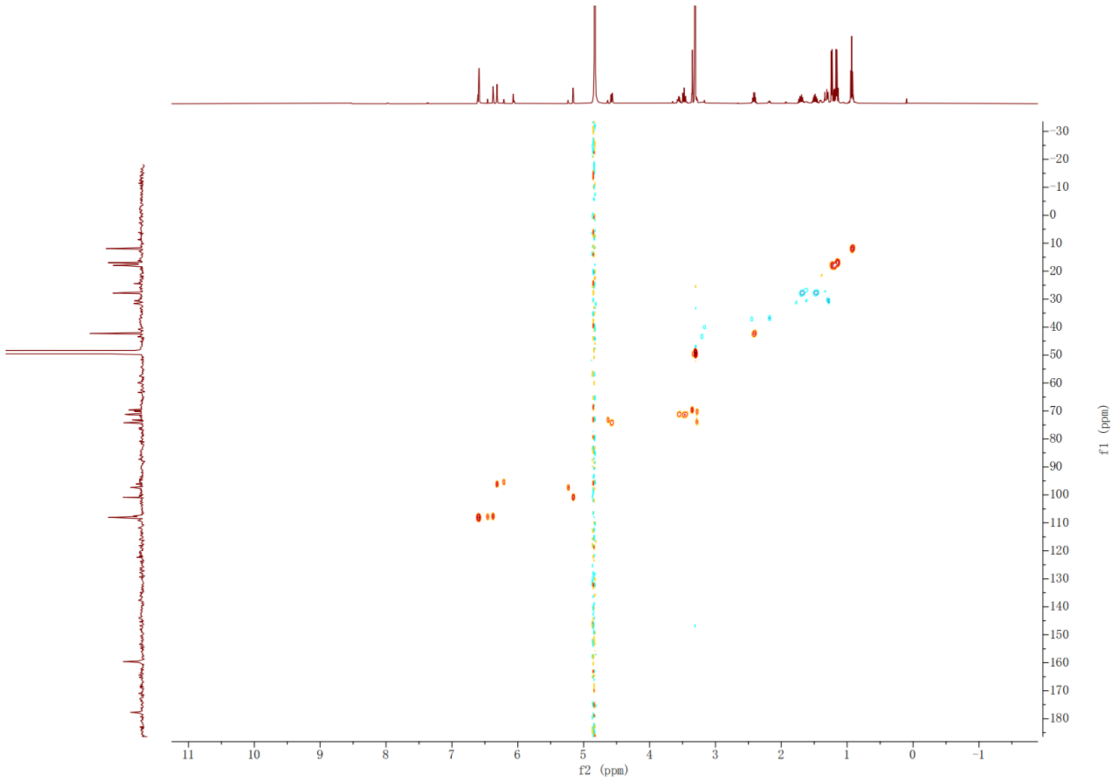

Fig. S14. HSQC spectrum of compound **2** (500 MHz, Methanol-*d*4).

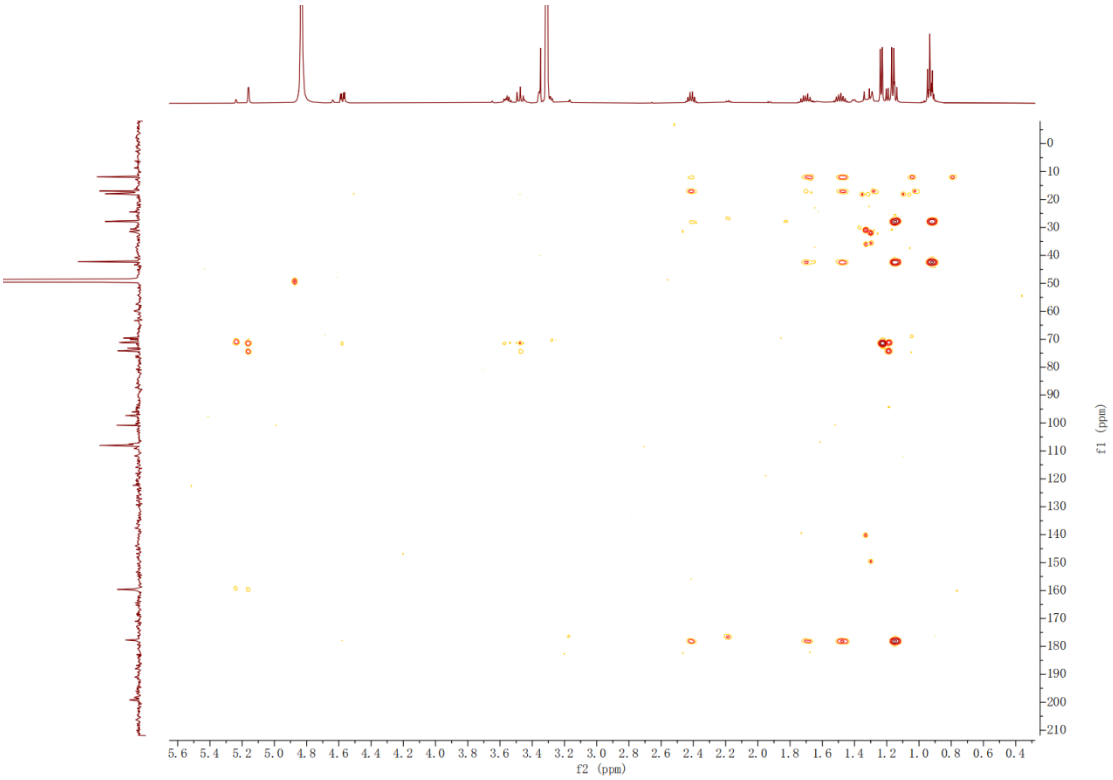

Fig. S15. HMBC spectrum of compound **2** (500 MHz, Methanol-d_4).

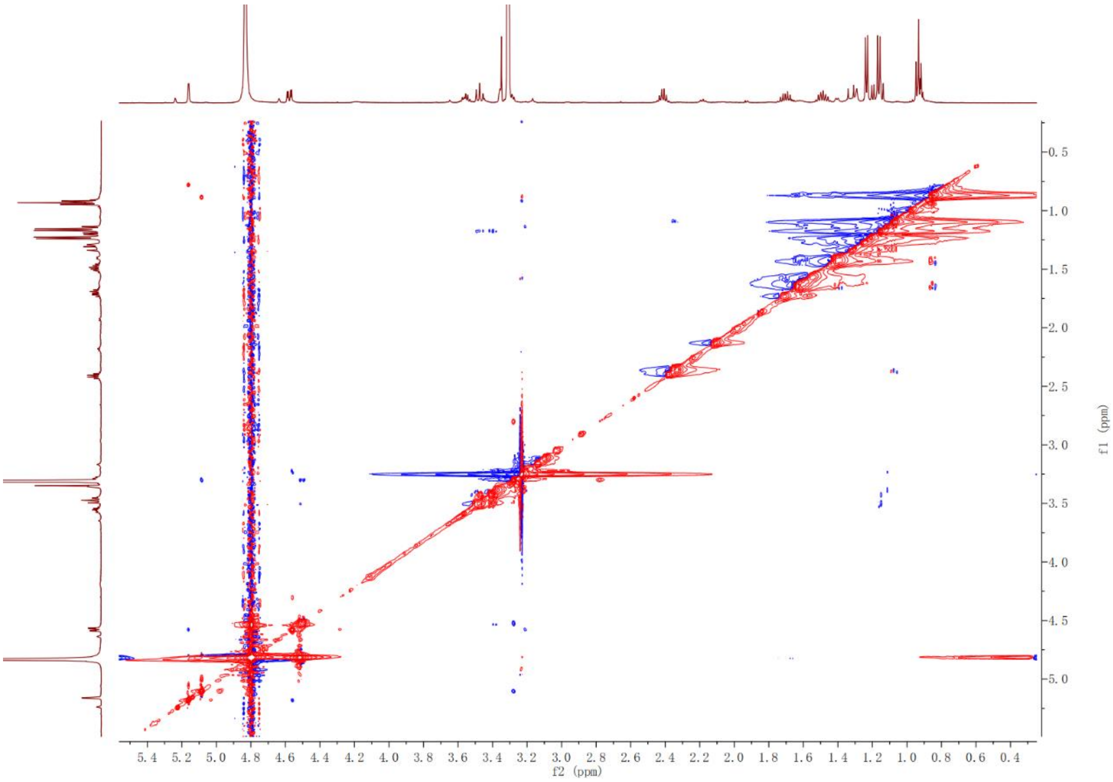

Fig. S16. ROESY spectrum of compound **2** (500 MHz, Methanol-d_4).

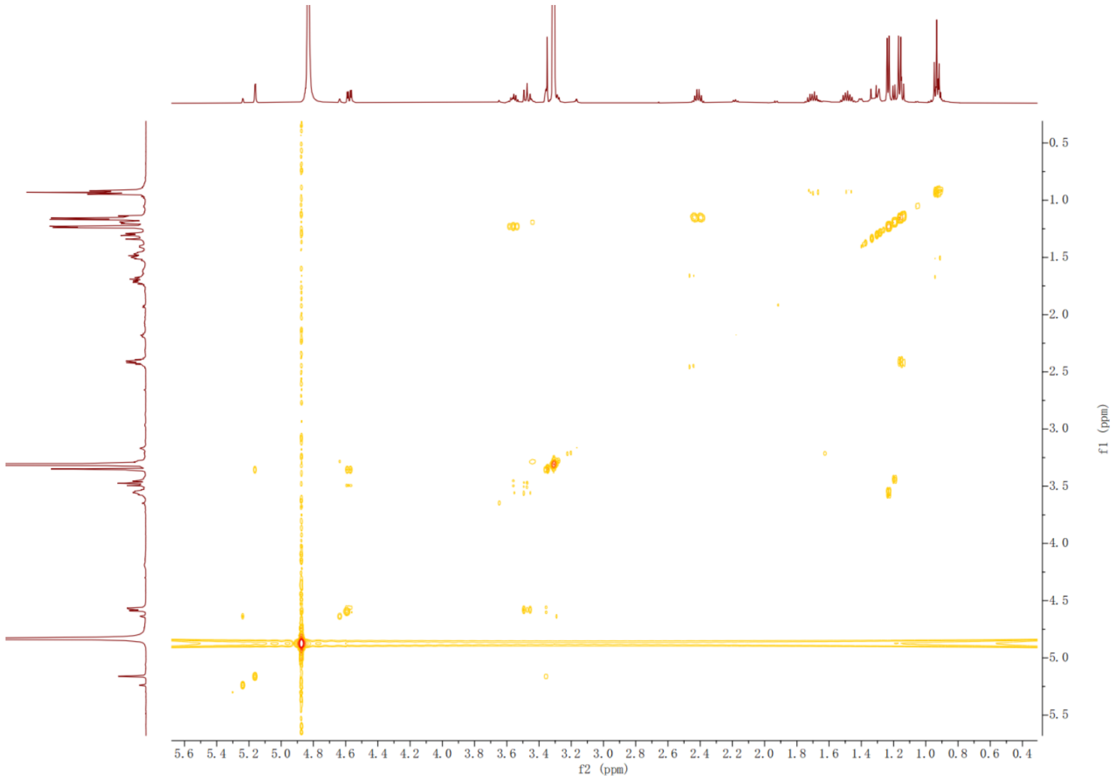

Fig. S17. ^{1}H-^{1}H COSY spectrum of compound **2** (500 MHz, Methanol-*d*4**).**
